# Effectiveness of a targeted telephone-based case management service on activity in an Emergency Department in the UK: a pragmatic difference-in-differences evaluation

**DOI:** 10.1186/s12913-022-08415-2

**Published:** 2022-08-15

**Authors:** Julian Elston, Felix P. Gradinger, Adam J. Streeter, Stephen Macey, Susan Martin

**Affiliations:** 1 Torbay and South Devon NHS Foundation Trust (TSDFT), Torbay, UK; 2 Community and Primary Care Research Group, Faculty of Health, University of Plymouth, Plymouth, UK; 3 Medical Statistics, Faculty of Health, University of Plymouth, Plymouth, UK; 4 Institute for Epidemiology and Social Medicine, University of Münster, Münster, Germany; 5 Planning and Performance, Torbay and South Devon, NHS Foundation Trust (TSDFT), Torquay, UK; 6 Quality Improvement, Torbay and South Devon, NHS Foundation Trust (TSDFT), Torquay, UK

**Keywords:** Frequent attenders, Artificial intelligence, Predictive models, Case management, Telephone-based Health coaching

## Abstract

**Background:**

This study evaluates the effectiveness of a targeted telephone-based case management service that aimed to reduce ED attendance amongst frequent attenders, known to disproportionately contribute to demand. Evidence on the effectiveness of these services varies.

**Methods:**

A 24-month controlled before-and-after study, following 808 patients (128 cases and 680 controls (41 were non-compliant)) who were offered the service in the first four months of operation within a UK ED department. Patients stratified as high-risk of reattending ED within 6 months by a predictive model were manually screened. Those positively reviewed were offered a non-clinical, nurse-led, telephone-based health coaching, consisting of care planning, coordination and goal setting for up to 9 months. Service effectiveness was estimated using a difference-in-differences (DiD) analysis. Incident rate of ED and Minor Injury Unit (MIU) attendances and average length of stay in intervention recipients and controls over 12 months after receiving their service offer following ED attendance were compared, adjusting for the prior 12-month period, sex and age, to give an incidence rate ratio (IRR).

**Results:**

Intervention recipients were more likely to be female (63.3% versus 55.4%), younger (mean of 69 years versus 76 years), and have higher levels of ED activity (except for MIU) than controls. Mean rates fell between periods for all outcomes (except for MIU attendance). The Intention-to-Treat analysis indicated non-statistically significant effect of the intervention in reducing all outcomes, except for MIU attendances, with IRRs: ED attendances, 0.856 (95% CI: 0.631, 1.160); ED admissions, 0.871 (95% CI: 0.628, 1.208); length of stay for emergency and elective admissions: 0.844 (95% CI: 0.619, 1.151) and 0.781 (95% CI: 0.420, 1.454). MIU attendance increased with an IRR: 2.638 (95% CI: 1.041, 6.680).

**Conclusions:**

Telephone-based health coaching appears to be effective in reducing ED attendances and admissions, with shorter lengths of stay, in intervention recipients over controls. Future studies need to capture outcomes beyond acute activity, and better understand how services like this provide added value.

## Background

The past decade has seen a steady rise in demand on Emergency Departments (ED) across the UK and internationally [1]. In the UK, ED admissions increased by 42% from 4.2 to 6.0 million between 2006–18, averaging 3.2% per year [2]. The largest increase has been in people aged 85 years or over (58.9%) [3].

It is estimated the increased demand is costing the National Health Service (NHS) £5.5 billion per year [3, 4], at a time when funding has not kept pace with historical investment rates [5], and placing significant pressure on EDs. The importance of reducing ED admissions is now a national priority, with its own NHS performance indicators [6].

However, the wide variation in attendance and admissions rates between sites suggests that many are avoidable [7]. NHS England have estimated that 40% of ED attendances and 24% of admissions are preventable [3, 4].

Studies suggest nearly two-thirds (61%) of ED attendances have at least one long-term condition (LTC) managed through continuous medication or treatment [2, 4]. For those attendances admitted for an overnight stay, one in three had five or more health conditions in 2015–16, up from one in 10 in 2005–06 [2, 4]. High intensity ED is also associated with high socio-economic deprivation and health inequalities and, across all age groups, it is linked with homelessness, unemployment, mental health conditions, drug and alcohol problems, criminality, and loneliness and social isolation [8].

One approach to reducing the rising tide of ED attendances and admissions has been to develop interventions that focus on frequent ED attenders [9]. This includes ambulatory care sensitive conditions that are purported to be prevented through provision of effective community care and pro-active case management [6]. It is theorised that care coordination, education on chronic conditions and coaching can support life-style changes and reduce social isolation, enabling patients to better self-manage their long-term conditions, as high use of acute and primary care services correlates with low patient activation [2]. Conversely, health coaching in people with long-term conditions has been shown to reduce use of health and social care services [10]. Frequent attenders are often defined as people who have visited an ED five or more times in the previous 12-month period [11], but this definition is not consistent across studies.

### Intervention context in UK

Evidence from two large Randomised Controlled Trials (RCTs) in Sweden [10], targeting patients attending ED at least 3 or more times in the previous 6 months (using an adaptive statistically-based screening strategy), suggests that combining case management with telephone-based health coaching has the potential to reduce emergency, in-patient and outpatient care [12]. In the last-year cohort of the study, in-patient admission rates fell by 15% and 23% compared to controls. However, statistically significant differences in ED attendance rates (a 7% fall) were only seen in the Zelen-design RCT (which randomises patients prior to consent [13]), more accurately mimicking effectiveness in a clinical setting [12]. Outpatient care, on the other hand, saw no effect and, in the traditional RCT, rates actually increased by a statistically significant 7%.

In the UK, there has been considerable interest in this intervention as a means of reducing ED demand, using a predictive model primed with historic secondary care activity data to identify high-risk patients. Similar to the Swedish trials, patients identified as high-risk subsequently undergo manual case note screening and, if assessed to have had avoidable health care episodes and are eligible, are offered the telephone-based health coaching service [12]; primarily a non-medical intervention (albeit nurse-led), with elements of care coordination, resources and support.

Torbay and South Devon NHS Foundation Trust (TSD NHS FT) was the first provider in the UK to commission this adapted service model, rather than opting to be part of an on-going UK-based RCT [14]. Although early non peer-reviewed results reported improved effectiveness on reducing ED activity [14], implementation of an intervention in a non-randomised population [15] could significantly reduce its effectiveness and impact [16]. This study sought to evaluate the effectiveness (rather than efficacy) of the service on ED activity in a real-world setting pragmatically, using existing activity data as a control group.

## Methods

### Setting

Torbay and South Devon is a coastal and moorland area in South West of England, UK. Its population is 293,400 with a high proportion of older people compared to England (1 in 4 residents are ≥ 65 years) with pockets of significant socio-economic need, particularly in Torbay.

### Study design

A 24-month controlled, before and after study, comparing patients electing to receive the intervention to those that did not (controls), followed up at 12 months after their service offer.

### Population

Patients attending ED between 1 October 2018 to 31 January 2019, aged 18 years or older, who were predicted to be at high risk of attending ED within the next 6 months were defined as frequent attenders. Prediction was based on a risk score provided by an automated predictive algorithm [16] primed with four years of historical of ED, in-patient and out-patient service use data as well as diagnosis codes, age, gender and discharge location. Risk scores were manually screened and selected daily by the service after assessing their potential to benefit from care coordination and health coaching (using their electronic hospital record). Typically, this took place within a week of their ED attendance.

As in the Swedish studies [12] and the UK-based RCT [14] the service’s exclusion criteria were: diagnosis of dementia, a psychotic disorder and mental disorder caused by alcohol or drug misuse, or with terminal cancer within the past 12 months; a life expectancy of < 1 year based on the prediction model; recent (within six months) or planned major surgery; severe hearing loss, language difficulties or cognitive ability that either require an interpreter or not sufficient for receiving and responding to telephone counselling; no access to a telephone or pregnant.

### Intervention

Intervention recipients were assigned a health navigator coach typically contacting them every 1–2 weeks by telephone and over a period of 6 to 9 months, during which they worked with the patient (using standardised templates) to optimise their medical treatment, nursing and care coordination, motivated them to improve their self-care and well-being (developing a joint plan with goal-setting). All members of the coaching team received two-day’s training, based on Reinius et al.*’s* [12] methodology, and used a bespoke software system and weekly case conferences to ensure consistency and timeliness of the intervention. Coaching focused on understanding each patient’s medical and social problems and what matters to them. The five-membered coaching team was nurse-led and, although comprised a paramedic and a physician in addition to three nurses, no medical advice was given.

### Data

Data was retrieved from the Trust’s ED IT system (Symphony) and Patient Administration System (PAS) between 1 October 2017 to 31 January 2020 for those offered the service. Extraction covered the period 12 months before and after each individual’s service offer date.

Field codes included date of birth, sex and postcode (to link to Index of Multiple Deprivation (IMD) 2019 rank [17]), GP code, dates of ED attendance and admission and Minor Injuries Unit (MIU) attendance, admission type (non-elective and elective), discharge date and destination (including death where available). Diagnosis codes were not available.

**Fig. 1:**
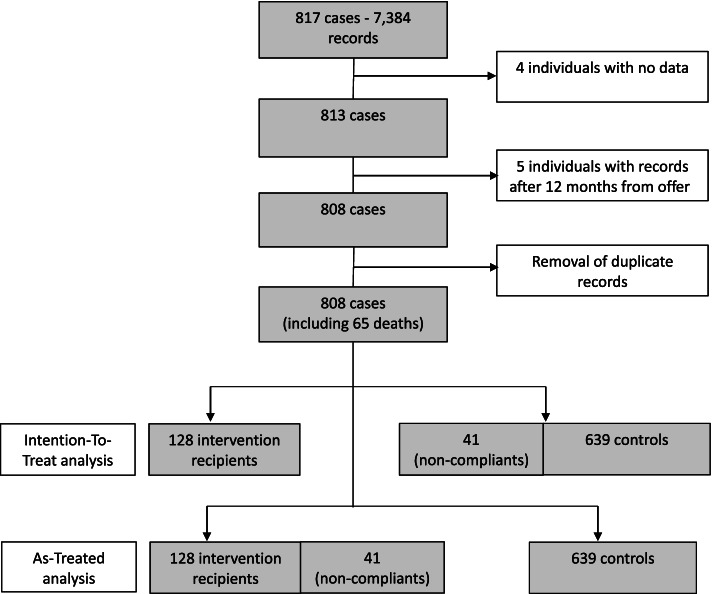
Data extraction, case categorisation and cohorts in the Intention-to-Treat and As-Treated Difference-in-Differences analyses

The initial search identified 817 people as offered the service within the first 4 months of operation. After removing duplicate entries and null records there were 808 people, 65 of whom died within 12 months of the service offer. Of the 808 offered the service, 128 actively took up the service offer after two invites, while 41 people who initially refused the service, subsequently took up a second possible offer after representing to ED within 12 months of their first offer. Therefore, in the Intention-To-Treat (ITT) analysis population, 128 were added to the service user group. Of the 680 controls, 41 (non-compliant controls) were considered to have received the intervention in the As-Treated analysis (see Fig. 1).

### Outcomes

Follow-up periods lasted 12 months in the period prior to offering the service, and 12 months after. Length of stay (days) was calculated by subtracting the discharge from the admission date for each admission, and summed for all admissions in each 12-month period before and after the service offer for each individual.

The primary outcomes were average rates of ED attendances and ED admissions (counts). Secondary outcomes were non-elective and elective admissions average length of stay (LOS) (days) and average rates of Minor Injury Unit (MIU) attendances. An MIU is a nurse-led, community-hospital based unit where highly-qualified and experienced nurses treat minor injuries between 8am-8pm daily. Although not specifically identified as an outcome by the commissioned service or the UK-based RCT [14], MIU data was included in the analysis as the local health system was promoting it as an alternative service for urgent non-life-threatening care.

### Analysis

A Difference-in-Differences (DiD) analysis estimated the intervention effect, while adjusting for a time-invariant confounding bias between the comparison groups, using the 12-month period prior to each participant’s follow-up period [18]. Estimation of the DiD effect in a regression model accommodated further adjustment of age at baseline and sex:

$${y}_{i,p}={\beta }_{0}+{\beta }_{t}.{T}_{i}+ {\beta }_{p}.{P}_{i}+{\beta }_{DiD}.{T}_{i}.{P}_{i}+{\beta }_{age}.age+{\beta }_{sex}.sex+ {\varepsilon }_{i}$$

where outcome y for the ith individual from the period p was regressed on indicators, P for period p and intervention T of the participant with estimated constant β_0_, in order to estimate the DiD coefficient, $\beta_{DiD}$. As the outcomes were count data and were zero-heavy, they were modelled by the negative binomial distribution, which also accommodated any over-dispersion. An offset modelled follow-up periods that were truncated because of death or had an additional day in a leap year. The DiD estimate for the coefficient, $\beta_{DiD}$, of the interaction term was then exponentiated to be interpreted as an Incidence Rate Ratio (IRR). Analysis was exploratory and interpretation of the results through 95% Confidence Intervals (CIs). Variation in ED attendance rates were also presented by sex, age band and IMD 2019 enabling an assessment of potential confounding by key demographic and socioeconomic characteristics. The complement of IRRs (1-IRR) was calculated to express effectiveness as a percentage.

## Results

Table 1 shows that only 15.8% (128/808) of positively screened patients took up the service offer, with a slightly greater proportion of females (56.7%) than males offered the service. The average age of participants was 75 years with a standard deviation of 15 year and ranging from 20 to 96 years. A greater proportion of females accepted the service offer (63.3%) compared to those that did not (55.4%). Intervention recipients were also younger compared to the controls on average (mean age: 69y versus 76y). There were no apparent differences between groups in the distribution of IMD quintiles with a greater percentage being in the first quintile, then third, then fourth. The largest proportion of the 41 controls, who switched to the intervention and were regarded as being in the service user group in the As-Treated analysis, were in the third IMD quintile. There was a slightly lower percentage of deaths in intervention recipients compared to controls (6.3% versus 8.4%) but not statistically different (*X*^2^ = 0.662; *p* = 0.415).

**Table 1:** Number and demographic characteristics of intervention recipients and controls

Characteristics		Intervention		Controls		Total
		recipients	Intention-to-Treat	As-Treated	Non-compliant	
N		128	680	639	41	808
Female: n(%)		81 (63.3)	377 (55.4)	357 (55.9)	20 (48.8)	458 (56.7)
Age: mean (sd) median [min, max]		69 (15) 71 [20,94]	76 (15) 79 [20,96]	76 (15) 79 [20,96]	74 (13) 77 [35,91]	75 (15) 78 [20,96]
Deprivation quintiles (IMD): n (%)	1—most	45 (35.2)	253 (37.2)	243 (38.0)	10 (24.4)	298 (36.9)
	2	14 (10.9)	55 (8.1)	46 (7.2)	9 (22.0)	69 (8.5)
	3	37 (28.9)	220 (32.4)	204 (31.9)	16 (39.0)	257 (31.8)
	4	22 (17.2)	81 (11.9)	80 (12.5)	1 (2.4)	103 (12.7)
	5—least	10 (7.8)	71 (10.4)	66 (10.3)	5 (12.2)	81 (10.0)
Deaths: n (%)		8 (6.3)	57 (8.4)	56 (8.8)	1 (2.4)	65 (8.0)

Key: *IMD* Index of Multiple Deprivation, *sd* Standard deviation

Table 2 shows that there were a greater average number of ED attendances and ED admissions among the intervention recipients group compared to the controls in the ITT and As-Treated (AT) populations. In the “after” period when the service was offered to the intervention group, there were falls in the average numbers of ED attendances and ED admissions for both intervention recipients and controls (ITT and AT). However, these falls were larger in intervention recipients compared to controls. In contrast, MIU attendance in intervention recipients was lower in the prior period and increased after receiving the service.

**Table 2:** Primary and secondary outcomes in Intervention recipients, Intention-to-Treat, As-Treated and non-complaint control cohorts

**Outcomes**	**Period**	**Intervention recipients**	**Intention-to-Treat**	**Controls**	**Non-compliant**	**Total**
				**As-Treated**		**All**
	**N**	**(*n* = 128)**	**(*n* = 680)**	**(*n* = 639)**	**(*n* = 41)**	***N* = 808**
		mean (sd) median [min, max]	mean (sd) median [min, max]	mean (sd) median [min, max]	mean (sd) median [min, max]	mean (sd) median [min, max]
ED and MIU attendances	Before	4.37 (5.28) 3 [ 0,43]	3.16 (3.62) 2 [ 0,57]	3.20 (3.72) 2 [ 0,57]	2.63 (1.51) 2 [ 0, 6]	3.35 (3.95) 2 [ 0,57]
(counts)	After	2.70 (3.69) 2 [ 0,24]	2.06 (2.95) 1 [ 0,28]	2.04 (2.96) 1 [ 0,28]	2.41 (2.97) 1 [ 0,14]	2.16 (3.09) 1 [ 0,28]
Difference in means		-1.67 (38.2%)	-1.10 (34.8%)	-1.16 (36.3%)	-0.22 (8.4%)	-1.19 (35.5%)
ED attendances (counts)	Before	4.21 (5.23) 3 [ 0,43]	2.92 (3.55) 2 [ 0,57]	2.96 (3.65) 2 [ 0,57]	2.27 (1.18) 2 [ 0, 5]	3.13 (3.89) 2 [ 0,57]
	After	2.46 (3.59) 1 [ 0,24]	1.91 (2.88) 1 [ 0,28]	1.90 (2.89) 1 [ 0,28]	2.12 (2.85) 1 [ 0,14]	2.00 (3.01) 1 [ 0,28]
Difference in means		-1.75 (41.6%)	-1.01 (34.6%)	-1.06 (35.8%)	-0.15 (6.6%)	-1.13 (36.1%)
MIU attendances (counts)	Before	0.16 (0.44) 0 [ 0, 2]	0.24 (0.75) 0 [ 0, 7]	0.23 (0.74) 0 [ 0, 7]	0.37 (0.94) 0 [ 0, 4]	0.23 (0.71) 0 [ 0, 7]
	After	0.23 (0.86) 0 [ 0, 8]	0.15 (0.51) 0 [ 0, 6]	0.14 (0.49) 0 [ 0, 6]	0.29 (0.75) 0 [ 0, 3]	0.16 (0.58) 0 [ 0, 8]
Difference in means		0.07 (-43.8%)	-0.09 (37.5%)	-0.09 (39.1%)	-0.08 (21.6%)	-0.07 (30.4%)
ED admissions (counts)	Before	2.68 (2.41) 2 [ 0,14]	2.10 (1.80) 2 [ 0,22]	2.12 (1.84) 2 [ 0,22]	1.83 (1.02) 2 [ 0, 5]	2.19 (1.92) 2 [ 0,22]
	After	1.73 (3.07) 1 [ 0,24]	1.48 (2.25) 1 [ 0,18]	1.48 (2.23) 1 [ 0,18]	1.46 (2.51) 0 [ 0,12]	1.52 (2.40) 1 [ 0,24]
Difference in means		-0.95 (35.4%)	-0.62 (29.5%)	-0.64 (30.2%)	-0.37 (20.2%)	-0.67 (30.6%)
ED admissions (emergency) LOS (days)	Before	3.49 (2.96) 3 [ 0,17]	2.60 (2.74) 2 [ 0,41]	2.63 (2.81) 2 [ 0,41]	2.10 (1.18) 2 [ 0, 6]	2.74 (2.80) 2 [ 0,41]
	After	1.93 (3.16) 1 [ 0,24]	1.60 (2.36) 1 [ 0,18]	1.61 (2.34) 1 [ 0,18]	1.59 (2.67) 0 [ 0,12]	1.66 (2.50) 1 [ 0,24]
Difference in means		-1.56 (44.7%)	-1.00 (38.5%)	-1.02 (38.8%)	-0.51 (24.3%)	-1.08 (39.4%)
ED admissions (elective^α^) LOS (days)	Before	0.73 (1.30) 0 [ 0, 8]	0.73 (2.43) 0 [ 0,40]	0.73 (2.49) 0 [ 0,40]	0.68 (1.21) 0 [ 0, 5]	0.73 (2.29) 0 [ 0,40]
	After	0.57 (1.05) 0 [ 0, 7]	0.68 (2.87) 0 [ 0,59]	0.70 (2.95) 0 [ 0,59]	0.39 (0.70) 0 [ 0, 3]	0.66 (2.66) 0 [ 0,59]
Difference in means		-0.16 (21.9%)	-0.05 (6.8%)	-0.03 (4.1%)	-0.29 (42.6%)	-0.07 (9.6%)

Key: *ED* Emergency Department, *MIU* Minor Injuries Unit, *LOS* Length of stay, *sd* Standard deviation; ^α^ = day case attendance at ED for tests, procedures, investigations typically admitted via the Acute Medical Unit (AMU) or Clinical Decision Unit (CDU) in ED

The average length of stay for ED admissions was higher in services users for emergency ED admissions than controls by less than a day, but not for elective admissions which were similar. Length of stay fell after the service offer for both emergency and elective admissions, although the reduction was greater in intervention recipients compared to controls (ITT and AT).

Table 3 shows that the IRR for intervention recipients to controls from the DiD analysis of the ITT sample was 0.856 (95% CI: 0.631, 1.160) for ED attendance with no admission and 0.871 (95% CI: 0.628, 1.208) for an admission following an ED attendance. However, the incidence of attendance in MIU was significantly greater in the intervention recipients, being over twice that of the controls (ITT IRR: 2.638 (95% CI: 1.041, 6.680)), having adjusted for the prior period. The confidence intervals for the DiD estimates of attendances other than those at the MIU included the null IRR of 1, and so could not be considered statistically significant. For all DiD results for admissions and ED attendances, the As-Treated estimates, though marginally closer to the null IRR value of 1, were not markedly different from the ITT estimates.

**Table 3:** Incident Rate Ratios and effectiveness estimates for outcomes in the Intention-to-Treat (ITT) and As-Treated analyses

Outcomes	Incidence Rate Ratio (95%CI)		Effectiveness, % (95%CI)	
	**Intention-to-Treat**	**As-Treated**	**Intention-to-Treat**	**As-Treated**
ED and MUI attendances (counts)	0.923 (0.687, 1.240)	0.980 (0.750, 1.280)	7.7 (31.3, -24.0)	2.0 (25.0, -28.0)
ED attendances (counts)	0.856 (0.631, 1.160)	0.918 (0.697, 1.210)	14.4 (36.9, -16.0)	8.2 (30.3, -21.0)
MIU attendances (counts)	2.638 (1.041, 6.680)^*^	2.093 (0.932, 4.698)	-163.8 (-4.1, -568.0)	-109.3 (6.8, -369.8)
ED admissions (counts)	0.871 (0.628, 1.208)	0.884 (0.657, 1.189)	12.9 (37.2, -20.8)	11.6 (34.3, -18.9)
ED admissions (emergency) LOS (days)	0.844 (0.619, 1.151)	0.870 (0.657, 1.153)	15.6 (38.1, -15.1)	13.0 (34.3, -15.3)
ED admissions (elective) LOS (days)	0.781 (0.420, 1.454)	0.702 (0.401, 1.229)	21.9 (58.0, -45.4)	29.8 (59.9, -22.9)

Key: *ED* Emergency Department, *MIU* Minor Injuries Unit, *LOS* Length of stay; ^*^ = statistically significant at 5% level

According to the summary data, the mean length of stay (LOS) following elective ED admission were similar in the prior period, but there was a greater overall reduction of 0.16 days among the intervention recipients compared to the reduction of 0.05 days among the ITT controls. For the emergency ED admissions the reduction in mean LOS was: 1.56 days for intervention recipients compared to 1.00 days for the controls.

The DiD analyses for LOS indicated a larger intervention effect on elective than emergency ED admissions, with mean LOS reduced by an estimated 21.9% (95%CI: 58.0, -48.4) and 15.6% (95%CI: 38.1, -15.1) respectively. The DiD estimates for the As-Treated sample indicated a slightly larger effect of the intervention on LOS following elective ED admission and a slightly weaker effect on LOS following emergency ED admission.

## Discussion

This study presents the findings from a service evaluation of a pro-active case-management intervention for frequent attenders in ED, using a pragmatic, controlled before-and-after design. It supports a growing body of observational and experimental evidence that case management approaches can help reduce ED attendance in frequent visitors to ED and associated, acute activity [19]. The findings show that, for this sample, the service appeared to reduce attendance rates at ED by 14–15% in services users over controls, although this impact was off-set slightly by an increase in MIU attendances. Intervention recipients also had a relatively larger fall in ED admissions over controls by 12–13% with their length of stay also falling by 15.6% and 21.9% in relation to elective and emergency admissions respectively. However, these findings were not statistically significant, possibly due to under powering (for example, the UK-based RCT estimated 1,853 patients were required to detect a similar effect at 90% power and α = 0.048 [14]). MIU attendances, on the other hand, saw an adjusted incidence rate double (statistically significant), albeit from a low prior rate. However, this represented a much lower number of attendances compared to ED. It is possible that intervention recipients had adjusted their behaviour following the intervention i.e. attending MIU instead of ED as their first point of call. Although not specifically identified as an outcome by the service provider, this suggests that the intervention may have shifted some of the activity and costs elsewhere in the health and social care system—an important consideration in future study design. The effect on admissions and LOS in emergency admissions were commensurate with that of ED attendances. However, as the effect on LOS for elective admissions was greater, this suggests the intervention was exerting an additional influence here.

### Comparison with existing evidence

However, the estimates of effectiveness were considerably less than the percentage fall in rates after the service offer, which was typically between 30–40% in both intervention recipients and controls across most outcomes. Since participants and their controls were selected according to the likelihood of future ED attendance, partly informed by past high ED attendance, the observed fall in the outcomes from the prior to the intervention period could be at least partly explained by regression to the mean, where treatment allocation is based on extreme values in the prior or pre-test period. This phenomenon has been reported in other studies with similar study designs [19, 20]. Our study, nevertheless, observed a differential effect of the intervention that was consistent across all the outcomes, albeit not significant statistically.

The estimates of effectiveness in this study were similar to the Swedish RCTs [12], if not slightly larger for ED attendances, suggesting that predictive modelling guided screening may be performing better than the previously-used adaptive, categorical-based approach to screening in Sweden.

Comparisons with other studies is challenging, not least as definitions of ‘frequent attenders’ are inconsistently used in studies [19, 21] ranging from 2 to ≥ 10 attendances within six months, or within a year [11, 22]. In this service, the identification process (a predictive algorithm generated risk score, plus manual screening) predominantly identified lower frequency attenders (the median was 3.95) and slightly older than some other studies [12, 21, 23], but not atypical for frequent attenders in England [33]. The selection criteria may have played a role as it excluded people with mental health and alcohol and drug misuse diagnoses, often high users of ED with complex needs [7], requiring specialist input or knowledge. These groups are associated with more positive study outcomes, although often requiring high intensity case management and multi-disciplinary team input [19, 23]. Given that the proportion of all ED attenders screening positive was 4–5% (service data) and only one in six postives took up the service, far less than in the Swedish RCT [12] (the reasons for this were not clear), the scope of this intervention to significantly impact on overall ED activity might be limited.

With the exception of Australian [24] and Swedish trials [12], other studies assessing pro-active case management interventions were based in the USA, and thus operating in a different health system context. Nevertheless, one of these studies also assessed a decision support programme to help identify high attenders [25]. A controlled before-and-after evaluation showed a larger effect than in our study, despite the service not including goal setting or coaching [25].

Predictive modelling and artificial intelligence algorithms are increasingly being used to predict high users of other services, for example, exacerbations in COPD [26] or risk of falls [27]. A recent review of case management interventions identified access to and close partnerships with local healthcare providers and community resources as key to their success [23], with some predictive models now factoring in the degree of integration of service providers [28], neighbourhood characteristics, and levels of socio-economic need [29]. Recent studies of ED attendance also confirm that socio-economic status, mental health and multiple comorbidities [2, 7, 30] often result in complex bio-psycho-social problems which, in turn, can lead to high attendance [31]. In this study, mortality was 8% (65/808), suggesting the algorithm’s prediction of life expectancy < 1 year was not strong, as mortality was higher than studies in Sweden (although not elsewhere [21]), and it may be picking up people for whom multiple healthcare attendances is appropriate. Thus, more multi-site research is required to identify what data may improve the specificity of identifying people at risk of re-attending ED as well as those that stand to benefit most from pro-active case management. This would likely improve the cost-effectiveness, impact and potential scalability of these services.

### Strengths and limitations of the study

This was a pragmatic, low cost, controlled before-and-after evaluation [32], based on clear selection criteria, with a reasonably-sized cohort and length of follow-up period [19]. As such, it reported on the effectiveness of the service in a real-world setting and cohort, rather than what might be reported from a sample of services users selecting to take part in an RCT.

Nevertheless, it was small relative to the size of the Swedish RCTs [12]. Evaluating the service beyond the first four months of operation, may have improved the study’s precision and performance of the predictive model (which also experienced some set-up problems), but was not possible due to commissioning arrangements and COVID-19 skewing outcomes data. While intervention recipients and controls also had slightly different characteristics in relation to sex and, in particular, age, a known predictor of ED attendance [33], these were controlled for in the analysis. However, as diagnostic codes were not available it is possible that the differences in outcomes seen could be explained by differential disease profiles (type or change in seriousness over time) between participant and controls, if the disease modifies the intervention effect [34]. The IRR estimates rested on the assumption that these and other characteristics between the two groups did not differ between the prior and prospective study periods. The DiD analysis also assumed that the trend in the intervention group would have been paralleled in the controls, had the intervention not been applied. This is an untestable assumption, although all lengths of stay and admission counts both exhibited a downward trend from the prior to the study period. An alternative approach to data analysis could be afforded by considering the times between ED attendances, rather than the frequency of these over time, and adjusting for confounding using a before-and-after method applicable to time-to-event data, the prior event rate ratio method [18]. However, as well as invoking some extra assumptions, this would also require further data on event times and by having to extend this to sequential events, the results may offer a less intuitive insight than those from the DiD design used in this study. Finally, the analysis assumed that there are no other interventions being implemented concurrently that might have confounded the study findings.

## Conclusion

Telephone-based health coaching using a predictive AI algorithm appears to be moderately effective in reducing ED attendances, admissions and length of stay, but has a small impact on overall ED activity. Studies and service evaluations need to consider historic patterns of frequent attendances in their design to provide realistic assessments of service impact and added value. Pragmatic, study designs using existing service data, can be helpful in informing policy, practice and academia and more appropriately reflect the implementation of policies and services under real-world conditions, albeit with the inherent limitations of the data.

## Data Availability

The dataset analysed in this study is not publicly available as this was a service evaluation and the Caldicott Guardian (Information Governance lead) at TSDFT only gave permission to process the data in a secure, anonymised format, not to share it.
